# Endoscopic Resection of Tailgut Cyst

**DOI:** 10.1155/2024/5538439

**Published:** 2024-06-11

**Authors:** Oleksandr Kiosov, Vladyslav Tkachov, Sergii Gulevskyi

**Affiliations:** ^1^Department of Faculty Surgery, Zaporizhzhia State Medical and Pharmaceutical University, Zaporizhzhia, Ukraine; ^2^Multidisciplinary Surgical Department, University Clinic of Zaporizhzhia State Medical and Pharmaceutical University, Zaporizhzhia, Ukraine

## Abstract

Tailgut cyst or retrorectal cystic hamartoma is a rare congenital lesion, thought to arise from a portion of the embryological hindgut, usually benign, with no or unspecific symptoms, mainly diagnosed in middle-aged women. Complete surgical resection of the cyst is recommended to avoid complications and confirm the diagnosis. In this report, we present our experience in the successful endoscopic management of a tailgut cyst, outlining the endoscopic resection technique and discussing under what conditions this approach may be applicable.

## 1. Introduction

Retrorectal cystic hamartoma, also known as tailgut cyst, is an infrequent development lesion, thought to originate from a portion of the embryological hindgut, which failed to regress [1]. Tailgut cysts are predominantly benign, symptomless in about half of the cases, and appear in a 3 : 1 female-to-male ratio mainly between the ages of 40 and 60 years [2, 3]. The foremost complications associated with retrorectal development cysts encompass secondary fistula formation due to infection and malignant transformation [4]. The full surgical excision of the cyst with complete removal of its wall is thus strongly recommended [5]. Among the reported cases, surgical treatment predominantly entailed open surgery, with several cases of laparoscopic and transanal endoscopic microsurgery (TEM) approaches [2, 3, 6]. Herein, we present the case of successful endoscopic management of small retrorectal cystic hamartoma.

## 2. Case Report

**Figure 1:**
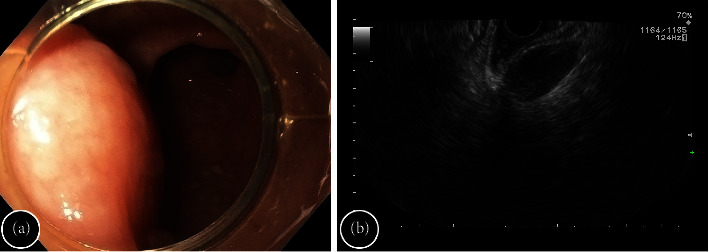
Diagnostic features of retrorectal cyst. (a) Colonoscopy reveals a hemispheric protrusion on the posterior wall of the rectum. (b) EUS showed a clearly defined hypoechoic mass measuring 25 × 13 mm.

A 45-year-old woman with a retrorectal tumor, revealed as an incidental finding by previous computed tomography, was hospitalized at the multidisciplinary surgical department with no complaints. There was no family history of gastrointestinal malignancy. On the endoscopy, a hemispheric bulge, 2 cm in diameter and covered with intact mucosa, was observed on the posterior rectal wall at 11 cm from the anal verge (Figure 1(a)). The instrumental palpation made by pressing the closed cold biopsy forceps down on the lesion showed a resiliently elastic consistency of the tumor and a free shift of mucosa above it. Due to contraindications for magnetic resonance imaging (MRI), endoscopic ultrasonography (EUS) was conducted, displaying a 25 × 13 mm oval-shaped tumor with a hypoechoic inhomogeneous content with a well-defined margin, close fitting to the posterior wall of the rectum, connected with its muscle layer (Figure 1(b)).

**Figure 2:**
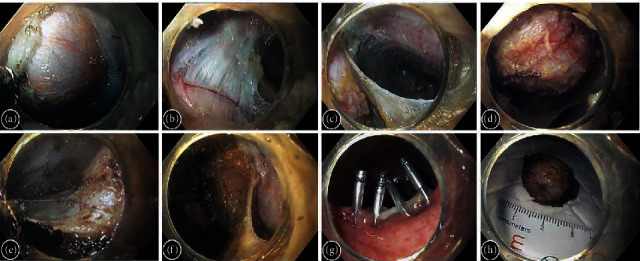
Retrorectal extraluminal endoscopic resection of tailgut cyst. (a) After dissecting the mucosa, submucosa, and circular muscle layer of the rectum, the cyst is visible. (b) Fibers of longitudinal muscle layer, attached to the upper part of the cyst capsule. (c) Rectal fascia, covering the cyst on the right side. (d) The cyst is partly separated from the surrounding tissues. (e) Dissection of dense band between loose connective tissues. (f) Tumor bed (on the left) after removal. (g) The mucosal incision was closed with 4 endoclips. (h) Macroscopic appearance of the lesion.

**Figure 3:**
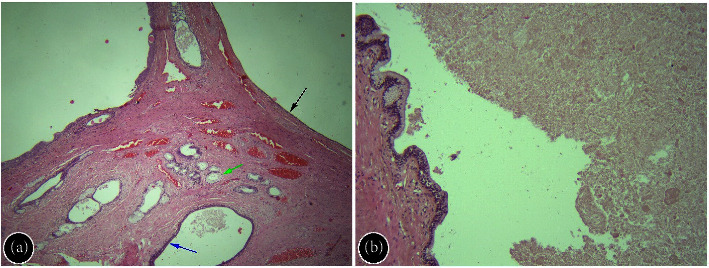
Microphotograph of tailgut cyst. (a) Multilocular structure, lined with transitional epithelium (blue arrow), serous epithelium (black arrow), and intestinal-type tall columnar mucinous epithelium with basally located nuclei (green arrow). The bundles of smooth muscle are visible within the stroma (H&E; x20). (b) The cystic wall (on the left) and mucous content (on the right) (H&E; x40).

Considering the close retrorectal location, absence of lymph node involvement, and lack of adjacent large blood vessels, the decision was made to proceed with retrorectal extraluminal endoscopic resection (REER) of the tumor. The operation was performed under general anesthesia using CO_2_ insufflation. We used the EVIS EXERA III GIF-HQ190 gastroscope with a distal transparent cap and a 2 mm ball-type endoscopic knife. After the submucosal injection of a 4% solution of succinylated gelatin dyed with indigo carmine, the mucosal incision was made 10 cm from the anus. Subsequently, a short submucosal tunnel was created, and thin, distended fibers of the internal muscular layer were visualized and dissected (Figure 2(a)). The tumor itself lay beneath, and its capsule was attached to the longitudinal muscular fibers (Figure 2(b)). The lesion was separated from the muscular layer, rectal fascia, and then from the fat and connective tissue of retrorectal space in a step-by-step manner (Figures 2(c) and 2(d)). Loose connective and adipose tissues were divided with denser bridges, connected to the cyst capsule (Figure 2(e)). The neoplasm was grabbed with an endoscopic loop and evacuated outward. The tumor bed was revised (Figure 2(f)). Blood vessels had been coagulated with a coagrasper. 4 endoscopic clips were applied for the ultimate closure of the mucosa (Figure 2(g)). The postoperative period was uneventful, and the patient was discharged in satisfactory condition on the fourth postoperative day. Macroscopically, the removed tumor looked like a round cystic neoplasm with a diameter of 2.5 cm (Figure 2(h)); on the cross section, the cavity was filled with a thick fluid of light brown color. Histology showed cystic hamartoma (tailgut cyst) (Figures 3(a) and 3(b)).

## 3. Discussion

Retrorectal tumors are rare, with a reported incidence of 1 per 40 thousand hospitalizations [7]. Two-thirds of them are cystic developmental lesions, which are classified into dermoid cysts, epidermoid cysts, teratomas, and enteric cysts. The latter include cystic rectal duplication and tailgut cysts, also called retrorectal cystic hamartomas [4, 8]. Currently, it is hypothesized that the etiology of tailgut cysts is linked to aberrant embryonic development, while the precise incidence in the general population remains unknown [5], by reason of absence of specific clinical symptoms, asymptomatic course in up to 50% of the cases [2], and heterogeneity of the cases presented. Although there is one reported case of nonsurgical treatment, where a tailgut cyst was confirmed with endoscopic ultrasound-guided fine-needle aspiration [9], along with a few instances of laparoscopic and TEM resections, the majority of cases are treated with open surgical approaches [3]. This is most likely due to the fact that the surgical approach is determined by size [10], and the larger the cyst is, the less space remains between the rectum and sacrum. In such a case, miniinvasive approaches become more complicated and preference is given to open surgery, considering that the complete resection of the lesion to prevent its recurrence is recommended. Moreover, it was shown by Hjermstad et al. [11] that the average maximal diameter of tailgut cysts is larger (4.6 cm) in symptomatic patients than in asymptomatic ones (3.2 cm). Chereau et al. [12] report a mean size of 5.4 cm with no correlation between size and symptoms of retrorectal tumors, though with a significant correlation between their size and malignancy, but only 28/47 tumors in the study were tailgut cysts. Other studies show the average diameter 4.1 cm [2] with the largest being 15 cm [11]. Meanwhile, endoscopic resection may be performed for subepithelial lesions less than 20 mm in size, with no mention of extraluminal lesions, in accordance with European guidelines [13], and for lesions less than 40 mm in size, following American guidelines [14]. To our best knowledge, this is the first reported case of endoscopic resection of tailgut cyst. We decided to treat the lesion endoscopically, due to its relatively small size, clear boundaries, location, that was adjacent to the rectal wall, and absence of large vessels around. Because of its extramural location, the mucosal bulge was not evident as seen in intramural lesions, making it challenging to determine the optimal location for the initial incision. Maintaining the dissection plane throughout the entire procedure was achievable, and no adverse events occurred during the procedure. On the other hand, the abovementioned size limits should be respected because it would be risky and technically demanding to work with a gastro- or colonoscope in a tight space with a cystic lesion larger than 4 cm. But for the small-sized retrorectal formations, endoscopic approach may be preferable, given its miniinvasiveness, since only the rectal wall and connective tissue need to be dissected to reach the lesion. Both transperineal and transabdominal surgical approaches seem to be associated with much greater tissue injuries, involving skin, muscles, vessels, and potentially pelvic or perineal nerves [5]. TEM shares similarities with the minimally invasive and well-visualized endoscopic approach we employed. It does not present drawbacks associated with open surgery and could be utilized for excising benign or carefully selected malignant retrorectal tumors with favorable outcomes and minimal complications [15–17]. However, documentation of TEM specifically for retrorectal tumors in the literature is sparse, and its availability among specialized medical facilities worldwide is limited. The presented REER technique employed bears resemblance to the submucosal tunneling endoscopic resection (STER) method, with the primary distinction being the necessity to separate the tumor from retrorectal fat rather than submucosal connective tissue. Endoscopists proficient in STER procedures should encounter no significant challenges in performing this procedure. Although we did not encounter any complications, it is essential to consider potential intraprocedural complications, such as major bleeding, the need for surgical conversion, or gas-related complications, given the nature of the technique employed and the anatomical region involved. The omission of endoscopic cases in literature can presumably be explained by the rarity of retrorectal tumors and their relatively large average size. We believe, that for small (<40 mm) lesions in retrorectal space, endoscopic resection could be considered as one of the treatment options.

## Data Availability

The data used to support the findings of this study are available from the corresponding author upon reasonable request.
